# A Systematic Approach to Mapping Recessive Disease Genes in Individuals from Outbred Populations

**DOI:** 10.1371/journal.pgen.1000353

**Published:** 2009-01-23

**Authors:** Friedhelm Hildebrandt, Saskia F. Heeringa, Franz Rüschendorf, Massimo Attanasio, Gudrun Nürnberg, Christian Becker, Dominik Seelow, Norbert Huebner, Gil Chernin, Christopher N. Vlangos, Weibin Zhou, John F. O'Toole, Bethan E. Hoskins, Matthias T. F. Wolf, Bernward G. Hinkes, Hassan Chaib, Shazia Ashraf, Dominik S. Schoeb, Bugsu Ovunc, Susan J. Allen, Virginia Vega-Warner, Eric Wise, Heather M. Harville, Robert H. Lyons, Joseph Washburn, James MacDonald, Peter Nürnberg, Edgar A. Otto

**Affiliations:** 1 Department of Pediatrics, University of Michigan School of Medicine, Ann Arbor, Michigan, United States of America; 2 Department of Human Genetics, University of Michigan School of Medicine, Ann Arbor, Michigan, United States of America; 3 Howard Hughes Medical Institute, University of Michigan School of Medicine, Ann Arbor, Michigan, United States of America; 4 Max-Delbrück Center for Molecular Medicine, Berlin, Germany; 5 Cologne Center for Genomics, University of Cologne, Cologne, Germany; 6 Institute for Genetics, University of Cologne, Cologne, Germany; 7 Department of Neuropaediatrics, Charite, Berlin, Germany; 8 Department of Pediatrics, Charite, Berlin, Germany; 9 University of Michigan Cancer Center, Ann Arbor, Michigan, United States of America; National Institute of Genetics, Japan

## Abstract

The identification of recessive disease-causing genes by homozygosity mapping is often restricted by lack of suitable consanguineous families. To overcome these limitations, we apply homozygosity mapping to single affected individuals from outbred populations. In 72 individuals of 54 kindred ascertained worldwide with known homozygous mutations in 13 different recessive disease genes, we performed total genome homozygosity mapping using 250,000 SNP arrays. Likelihood ratio Z-scores (ZLR) were plotted across the genome to detect ZLR peaks that reflect segments of homozygosity by descent, which may harbor the mutated gene. In 93% of cases, the causative gene was positioned within a consistent ZLR peak of homozygosity. The number of peaks reflected the degree of inbreeding. We demonstrate that disease-causing homozygous mutations can be detected in single cases from outbred populations within a single ZLR peak of homozygosity as short as 2 Mb, containing an average of only 16 candidate genes. As many specialty clinics have access to cohorts of individuals from outbred populations, and as our approach will result in smaller genetic candidate regions, the new strategy of homozygosity mapping in single outbred individuals will strongly accelerate the discovery of novel recessive disease genes.

## Author Summary

Many childhood diseases are caused by single-gene mutations of recessive genes, in which a child has inherited one mutated gene copy from each parent causing disease in the child, but not in the parents who are healthy heterozygous carriers. As the two mutations represent the disease cause, gene mapping helped understand disease mechanisms. “Homozygosity mapping” has been particularly useful. It assumes that the parents are related and that a disease-causing mutation together with a chromosomal segment of identical markers (i.e., homozygous markers) is transmitted to the affected child through the paternal and the maternal line from an ancestor common to both parents. Homozygosity mapping seeks out those homozygous regions to map the disease-causing gene. Homozygosity mapping requires families, in which the parents are knowingly related, and have multiple affected children. To overcome these limitations, we applied homozygosity mapping to single affected individuals from outbred populations. In 72 individuals with known homozygous mutations in 13 different recessive disease genes, we performed homozygosity mapping. In 93% we detected the causative gene in a segment of homozygosity. We demonstrate that disease-causing homozygous mutations can be detected in single cases from outbred populations. This will strongly accelerate the discovery of novel recessive disease genes.

## Introduction

The primary causes of most pediatric diseases remain elusive. However, it is becoming apparent that many pediatric diseases may represent recessive single-gene (monogenic) disorders. As an example, it was recently recognized that up to 25% of all cases with steroid resistant nephrotic syndrome (SRNS) in childhood are caused by recessive mutations of the *NPHS2* gene, although SRNS had not previously been viewed as a genetic disorder [1]–[4].

**Figure 1:**
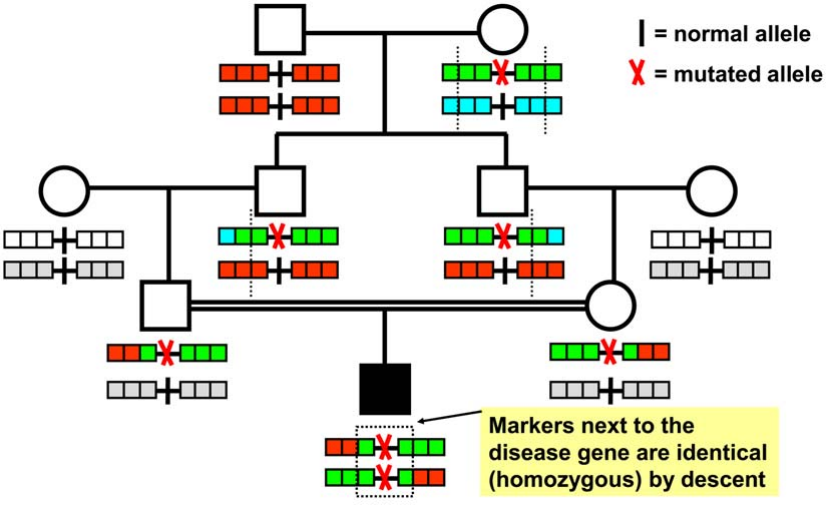
Homozygosity mapping of recessive disease genes. An individual with an autosomal recessive disease (filled symbol) whose parents are consanguineous will very likely be homozygous (identical) by descent for the disease allele (“mutated allele”), because a rare mutation may segregate from a common ancestor to the child through both the father's and mother's line, rendering the child homozygous for the mutation. Chromosomal segments surrounding the disease gene locus are shown here with 3 marker positions to either side. Different marker alleles are represented by different colors. Although for each parent-offspring succession there is an opportunity for a crossing over (dotted line) to occur in the parents' gametes, there is a high likelihood that in the affected child consecutive markers surrounding the mutation will not have recombined and will be identical (homozygous) by descent. This segment of homozygous markers (dotted box) can be detected as a “cZLR” peak in multipoint evaluation (see Figure S1 and Figure S2), leading to successful mapping of the disease gene. Note also that more remote consanguinity will give more opportunities for crossing over to occur and will thereby lead in the affected individual to fewer and shorter homozygous intervals that contain the disease gene (see Figures S2 and S3).

**Figure 2:**
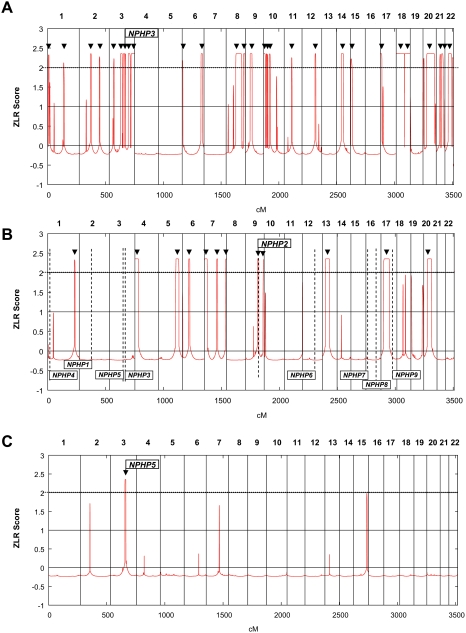
Homozygous mutations in recessive disease genes are positioned in cZLR peaks. Homozygosity profiles (see Figure S1 and Methods) are shown for individuals who have a known homozygous mutation in a recessive disease gene causing nephronophthisis (NPHP). Individuals are (A) from a consanguineous marriage, (B) without known consanguinity but originating from a geographic population with frequent consanguinity, and (C) originating from an outbred population. Non-parametric LOD scores were calculated with ALLEGRO assuming first-degree cousin consanguinity of the parents, regardless of actual consanguinity status. ZLR scores (minor allele frequency >0.3) were plotted over genetic distance across the genome, where chromosomal positions are concatenated from p- to q-arm (left to right). cZLR peaks (arrow heads) exceeding the empirical cut-off value of 2.0 (dotted line) as described in Figure S1 reflect possible segments of homozygosity by descent, one of which harbors the homozygous disease gene mutation in each patient (arrows with flags indicate the position of the mutated gene). (A) Individual A14-2 exhibits 28 cZLR peaks. This individual has a known homozygous *NPHP3* mutation (flagged position) and is from a consanguineous Venezuelan kindred with 17 documented consanguinity loops [16]. (B) Individual A8-1 exhibits 12 cZLR peaks. This family is not aware of consanguinity but originates from a geographic region where consanguinity is frequent (Turkey). Note that the homozygosity profile permits exclusion of homozygous mutations from 8 different *NPHP* loci (*NPHP1* and *NPHP3-9*), which are marked by vertical dashed lines flagged with gene name at the bottom, and directs mutation analysis to *NPHP2*, in which the individual carried the disease-causing homozygous mutation. (C) Individual F399-1 exhibits only 1 cZLR peak of 8.25 Mb width, which harbors the disease causing homozygous mutation of *NPHP5*. This individual originates from a geographic region where consanguinity is rare (Germany).

Progress has been made in unraveling the primary causes (etiologies) of pediatric disorders by identifying recessive disease genes using positional cloning by homozygosity mapping [5]. Homozygosity mapping is performed by total genome scan with polymorphic markers in individuals with a recessive disease whose parents are related. It tests the assumption that a homozygous mutation in a recessive disease gene is “identical by descent (IBD)” by segregating twice to the affected child from a common ancestor through both the maternal line and the paternal line (Figure 1). In addition, homozygosity mapping supposes that a short chromosomal segment surrounding the homozygous mutation has not been recombined by crossing over and that SNP (single nucleotide polymorphism) markers in a segment surrounding the mutation will therefore also be homozygous by descent (Figure 1) [5]. This short segment of homozygosity by descent can then be detected by multipoint homozygosity mapping as a “ZLR peak” (Figure 2) and will harbor the sought-after disease gene.

Gene identification by positional cloning holds multiple promises: i) It directly identifies the primary cause of a disease, thereby providing a secure starting point for the elucidation of the related pathogenesis. ii) Positional cloning often leads to the discovery of novel genes, revealing new mechanisms of cell physiology and development. iii) Since recessive single-gene disorders exhibit the strongest possible relationship between disease cause and disease phenotype, leading to a phenotype in virtually all individuals with mutations, they provide the most useful objective for molecular genetic diagnostics [6].

The number of causative genes for autosomal recessive disorders that are yet to be discovered is most likely in the thousands [6]. However, more rapid discovery of recessive disease genes is hampered by the lack of suitable consanguineous families with multiple affected children. In addition, mapping has resulted in too many putative loci that contain too many candidate genes for efficient gene identification.

Considering these impediments to gene discovery, we tested the hypothesis that SNP arrays may systematically allow gene identification by homozygosity mapping in single individuals from non-consanguineous (“outbred”) populations. If this were possible, it would offer the following important advantages: i) For any given disease phenotype, pediatric specialty clinics have access to far more patients who are from outbred populations and represent single cases than to patients who are from consanguineous background or represent sibling cases. ii) Homozygosity mapping in individuals who are from outbred populations but bear homozygous disease gene mutations by descent from an unknown distant ancestor may provide a single genomic candidate region small enough to allow successful gene identification by mutation analysis.

## Methods

### Subjects

We obtained blood samples and clinical and pedigree data following informed consent from individuals with two different groups of autosomal recessive kidney diseases, nephronophthisis (NPHP) or steroid resistant nephrotic syndrome (SRNS). Human subjects research was approved by the University of Michigan Institutional Review Board. The diagnoses were made by nephrologists based on standardized clinical criteria [7],[8]. Renal biopsies were evaluated by renal pathologists. Clinical data were obtained using standardized questionnaires (www.renalgenes.org).

### Mutation Analysis

In 1,069 families with a NPHP-like phenotype, who were referred to us for mutation analysis from worldwide sources over the course of 16 years, we performed mutation analysis by direct exon sequencing in the genes *NPHP1* through *NPHP9*, *AHI1*, and *MKS3*. Additionally, in 404 families with steroid-resistant nephrotic syndrome (SRNS), who were referred to us worldwide for mutation analysis over the course of 8 years we performed mutation analysis by direct exon sequencing in the genes *NPHS1* (nephrin) and *NPHS2* (podocin).

### Homozygosity Mapping

We performed genome-wide scans for linkage in 72 individuals from 54 families, who had a homozygous mutation in any of the 13 genes that cause NPHP or SRNS (*NPHP1* through *NPHP9*, *AHI1*, *MKS3*, *NPHS1* or *NPHS2*). We used single nucleotide polymorphism (SNP) arrays (GeneChip) from Affymetrix, Inc. Most individuals (n = 40) were examined at 250 K resolution (Human Mapping 250 K *Sty*I Array), 9 families at 50 K resolution (50 K *Hin*d Array), and 5 families at 10 K resolution (50 K *Xba* Array). Samples were processed, hybridized, and scanned using the manufacturer's' standard methods at the University of Michigan Core Facility (www.michiganmicroarray.com). As non-parametric likelihood ratio z-scores (ZLR scores) provide a good estimate of excess allele sharing [9], and since the GENEHUNTER [10] NPL score is conservative [11], we used the ZLR score implemented in ALLEGRO [12] to detect narrow segments of homozygosity. Parameters were set for a disease allele frequency of 0.001, and marker allele frequencies for Caucasians as specified by Affymetrix. We calculated ZLR scores under the hypothesis of consanguinity, using a standard pedigree structure first cousin marriage for parents of affected individuals. For single affecteds a non-existent sibling was included to enable non-parametric ALLEGRO runs. ZLR scores were plotted over genetic distance across the entire human genome using the GNUPLOT software (http://www.gnuplot.info/). In this way, maxima of ZLR scores, “ZLR peaks”, are expected to reflect segments of homozygosity by descent, which may harbor the homozygous mutation of the recessive disease gene. In the following we will refer to the ZLR plots as “homozygosity profiles” as described in Figure S1.

Using ALOHOMORA [13], ZLR scores were calculated using one marker every 100,000 nucleotides of human genomic sequence under three different conditions, i.e. for minor allele frequencies of >0.2, >0.3, and >0.4, respectively (Figure S1). In a small cohort of patients with known homozygous disease-causing mutations in *NPHS2* we established that ZLR peaks, which exceed the value of 2.0 under two out of the three conditions (minor allele frequencies of >0.2, >0.3, and >0.4), did in fact contain the known homozygous disease-causing mutation in this ZLR peak and exhibited continuous segments of homozygosity upon haplotype inspection. We therefore refer to peaks that fulfill these criteria as “consistent ZLR peaks” (“cZLR” peaks) (Figure S1).

All ZLR peaks of homozygosity considered “consistent” under these criteria were also present when using the criterion of 0.1 as cut off for minor allele frequency.

We later confirmed that in 67 of 72 cases (93%) the homozygous mutations in the causative gene were positioned within a cZLR peak (see Results). Thus, cZLR peaks can be utilized to indicate the position of homozygous mutations in recessive disease genes with high likelihood. We demonstrate below that cZLR peaks very rarely occurred in negative control individuals without homozygous mutations (see Results).

### Data Bases

Physical distance for SNP markers: genome browser, human, May 2004 at http://genome.ucsc.edu

## Results

### Homozygous Mutations Are Frequent in Rare Recessive Diseases

Homozygosity mapping can only be applied to individuals that bear homozygous mutations of recessive disease genes. Therefore, we first evaluated whether in a significant fraction of patients with mutations in 13 different recessive disease genes the disease was caused by homozygous rather than compound heterozygous mutations. We chose 9 different genes (*NPHP1-9*) [14] that cause the recessive kidney disorder nephronophthisis (NPHP), 2 genes (*AHI1* and *MKS3*) causing NPHP-like phenotypes, and 2 genes (*NPHS1* and *2*) that cause steroid resistant nephrotic syndrome (SRNS) [2], as we had access to DNA from worldwide cohorts of patients with these disorders. NPHP is the most frequent genetic cause of terminal kidney failure in children and young adults. Positional cloning of nine responsible genes (*NPHP1-9*) has revealed NPHP as a “ciliopathy”, relating its pathogenesis to dysfunction of primary cilia signaling [14].

In a worldwide cohort of 1,069 families with NPHP we detected the disease-causing mutation in 320 (30%). We excluded from the analysis 224 families with large deletions of *NPHP1*, as they are due to a unique mechanism of genomic DNA rearrangement [15]. In the remaining 96 families, the fraction of homozygous mutations for each gene was as follows: *NPHP1* (2/2; 100%), *NPHP2* (6/11; 55%), *NPHP3* (4/6; 66%), *NPHP4* (15/20; 75%), *NPHP5* (22/26; 85%), *NPHP6* (3/10; 30%), *NPHP7* (1/1; 100%), *NPHP8* (6/6; 100%), *NPHP9* (1/1; 100%), *AHI1* (2/5; 40%), and *MKS3* (5/8; 63%). Thus, the fraction of homozygous mutations for the 11 different NPHP-causing genes together was 70% (67/96) and ranged from 30–100%. The other patients had compound heterozygous mutations in these genes. In patients with NPHP there was some bias towards selection of consanguineous kindred. However, we have also ascertained a worldwide cohort of 404 families with SRNS for mutation analysis in the genes *NPHS1* and *NPHS2* without selection for consanguineous kindred. In 73/404 (18%) of these families we detected both mutated alleles of *NPHS2*. Thirty-three of the 73 (45%) had homozygous mutations [4]. *NPHS1* mutations were detected in 36 of 86 (42%) families with congenital nephrotic syndrome. Twenty-four of the 36 (67%) had homozygous mutations (Heeringa, unpublished). We thus demonstrate that for 13 different recessive disease genes a very high fraction (124/205; 60%) had homozygous mutations, although ascertainment was mostly by self-recruitment for mutation analysis and not specifically directed at patients from a consanguineous background. We conclude that in a large proportion of patients from worldwide cohorts with mutations in various rare recessive disease genes homozygosity mapping is potentially feasible.

### Homozygous Mutations Map to Homozygosity Peaks

We next tested the assumption that homozygous mutations of recessive disease genes are virtually always localized in segments of contiguous homozygous SNP markers derived from a (distant) common ancestor and can therefore be located by homozygosity mapping. The rationale of homozygosity mapping is summarized in Figure 1. In addition, we wanted to determine the shortest detectable size of homozygous segments as short segments will reduce the number of candidate genes. From our worldwide cohort of 124 families with known homozygous mutations in 13 different recessive genes (*NPHP1-9*, *AHI1*, *MKS3*, *NPHS1* and *NPHS2*) we selected for total genomes scan 72 patients from 54 different families that had sufficient DNA available. The selected families represented the entire spectrum of consanguinity from first degree cousin relations to outbred populations. Families, patients, and their homozygous mutations are listed in Table S1.

Following total genome search by homozygosity mapping primarily using 250 K SNP arrays we plotted for each individual “homozygosity profiles” across the genome as defined in Figure S1. Examples are given in Figure 2 for three individuals with homozygous mutations in *NPHP3*, *NPHP2*, and *NPHP5*, respectively. Homozygosity plots of all 72 cases are available from the authors. Generation of homozygosity profiles suggested that the number of cZLR peaks per genome reflected the degree of consanguinity of an individual (Figure 2). For example, individual A14-2 who is from a consanguineous Venezuelan kindred with 17 documented consanguinity loops, exhibited 28 cZLR peaks (Figure 2A), whereas individual A8-1, who is not aware of consanguinity but originates from a geographic region with frequent consanguinity (Turkey) exhibited 12 cZLR peaks (Figure 2B). Only one of the 12 cZLR peaks colocalized with one of the 9 different *NPHP* loci. This was the *NPHP2* locus, in which the patient has a homozygous disease causing mutation (Figure 2B). This demonstrates that in diseases with multiple responsible loci the generation of a homozygosity profile can help select the locus relevant for mutation analysis from a high number of alternative disease loci. Thus, homozygosity profiles will reduce the number of exons to be examined in molecular genetic diagnostics. In the example given it was reduced from 220 exons for *NPHP1* through *NPHP9* to 16 exons for *NPHP2* only. In contrast, individual F399-1, who originates from a geographic region where consanguinity is rare (Germany) exhibited only one single cZLR peak of 8.2 Mb width, which harbored the disease-causing homozygous mutation in *NPHP5* (Figure 2C). These data gave a first indication that the number of ZLR peaks per genome reflect the degree of consanguinity and that homozygous mutations in outbred populations can be localized to a single cZLR peak that is unique to an individual's genome.

The strategy of homozygosity mapping carries the theoretical risk that the mutation in the recessive disease gene may not be detected in a homozygosity peak. In order to test whether homozygous disease-causing mutations were practically always positioned in cZLR peaks, we generated homozygosity plots as defined in Figure S1 (see also Figure 2) of all 72 individuals from 54 families with homozygous mutations in one of the 13 different recessive genes tested. We found that in 67 of 72 cases (93%) the homozygous mutation of the causative gene was in fact positioned within a cZLR peak (data available from the authors). In the five individuals with absence of a cZLR peak the mutation was embedded in a homozygous haplotype too short for detection (see below). In the 13 rare recessive disease genes studied, we thus confirmed the hypothesis that most homozygous mutations (93%) can be mapped to a cZLR peak.

### The Number of cZLR Peaks Reflects Consanguinity

**Figure 3:**
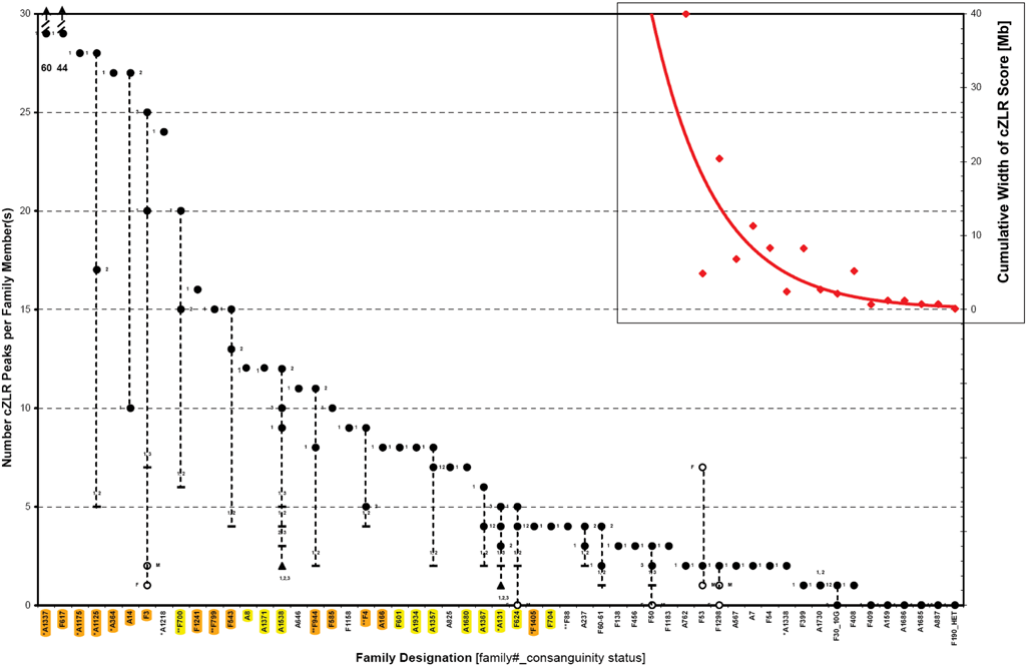
Detection limit for recessive disease loci in 72 affected individuals of 54 families with known homozygous disease-causing mutations. Total genome search by homozygosity mapping was performed by typing 250 K SNP arrays in 72 individuals of 54 families with known homozygous mutations in 13 different autosomal recessive kidney disease genes. Under the hypothesis of identity by descent, total genome homozygosity profiles were calculated as described in Methods. The numbers of cZLR peaks (Figure S1) were plotted per family. Families were ordered from the highest to the lowest number of cZLR peaks from left to right. On the X-axis orange highlighting is used for 15 families with known consanguinity, yellow highlighting for 12 families with no known consanguinity but originating from a population with frequent consanguinity, and no highlighting (white) for 27 families from outbred populations. Family number is preceded by one or two asterisks if data were generated by 50 K or 10 K SNP arrays, respectively. Filled circles denote affected individuals with homozygous mutations (siblings numbered as 1, 2, or 3 to the left or right), open circles denote father (F) or mother (M). Dashes denote number of peaks for two affected siblings calculated together (individual numbers shown above dashes), filled triangles for three siblings calculated together (individual numbers shown below triangle). Note that families with known consanguinity (orange) cluster in the left third of the plot with cZLR peaks ranging from 60 to 8, families without known consanguinity but from a populations, in which consanguinity is frequent (yellow) cluster in the middle with number of cZLR peaks ranging from 8 to 4, whereas families from outbred populations (no highlighting) cluster in the right third of the plot with cZLR peaks ranging from 4 to 0. Inset: In families from outbred populations that yielded 2 or fewer cZLR peaks, cumulative physical width in Mb for all cZLR peaks together is plotted (red, with axis on the right; exponential fit; if no peak was detected extent of homozygous markers at the gene locus is given). The cut off at which no cZLR peak was detectable was for a cZLR peak width of 2.1 Mb (see also Table 1). Note that parents of family F30_10G are known to be related 10 generations back, and that F190_HET represents an outbred control individual with a known compound heterozygous mutation in *MKS3*.

To examine the relationship between the number of cZLR peaks and degree of consanguinity we plotted the number of cZLR peaks for each of the 72 individuals from 54 families with homozygous disease-causing mutations (Figure 3). The number of cZLR peaks ranged from 60 to 0 (Figure 3). When we sorted families from left to right from highest to lowest number of cZLR peaks, the number of cZLR peaks reflected the degree of inbreeding in each family as follows (Figure 3): i) Families with known consanguinity (orange highlighting in Figure 3) clustered in the left third of the plot with the number of cZLR peaks ranging from 60 to 8. Fourteen of fifteen consanguineous families (orange) had 8 or more cZLR peaks. The highest number of cZLR peaks was seen in the following closely inbred families: A1337 (1^st^ degree cousins, Turkey), F617 (Native American), A1175 (1^st^ degree cousins, Turkey), A1125 (known consanguinity, Turkey), A364 (known consanguinity), A14 (17 consanguinity loops, Venezuela), and F3 (1^st^ degree cousins) [16]. ii) Families without known consanguinity, but originating from geographic regions where consanguinity is frequent (yellow highlighting in Figure 3), clustered in the middle third of the plot with, 8 of 12 families having 8 to 4 cZLR peaks. These families originated mostly from Turkey and Arab countries (Table S1). iii) Outbred families, i.e. families without known consanguinity and originating from geographic regions where consanguinity is rare (no highlighting in Figure 3), clustered in the right third of the plot with cZLR peaks. Twenty-three of the 27 outbred families had 4 to 0 cZLR peaks (0 denoting that no cZLR peak was detected). These families originated mostly from Western Europe and the US (Table S1). No family from a consanguineous population had 3 or less cZLR peaks.

We thereby demonstrated through graphical evaluation of total genome homozygosity profile data, that the number of cZLR peaks of individuals reflected their degree of inbreeding and thereby the extent of homozygosity by descent. Specifically, the number of homozygosity peaks was 8–60 in families with known consanguinity, 4–8 in families with unknown consanguinity but originating geographic background “at risk” for consanguinity, and it was 0–4 in families from outbred populations. Homozygosity profiles can therefore be viewed as “consanguinograms” that reflect the degree of inbreeding of an individual. The data set generated (Figure 3) gave us the opportunity to examine the limit of resolution for detecting short segments of homozygosity in single individuals with homozygous disease-causing mutations from outbred populations.

### Homozygous Disease Mutations Can Be Mapped in Single Individuals of Outbred Populations

Until now, successful gene identification by homozygosity mapping has been mostly based on consanguineous kindred that have multiple affected individuals. Close consanguinity of such kindred generate homozygous segments broad enough to be detected by SNP marker sets of low density (e.g., 50 K marker set). The presence of multiple siblings or cousins helps refine the candidate region to fewer cZLR peaks that overlap between affected siblings or cousins. For example, the number of 9, 12 and 10 cZLR peaks in siblings A1538-1, -2, and -3, respectively is reduced to 3, 4, and 5 cZLR peaks if each pair of siblings is evaluated together and to 2 cZLR peaks if all three sibs are evaluated together (Figure 3). However, a severe limitation to successful positional cloning of recessive disease genes by homozygosity mapping lies in the fact that sufficient numbers of consanguineous kindred with multiple affecteds and mutation of the same gene are very hard to ascertain. In our 16-year experience in worldwide recruitment of 1,069 patients with NPHP, non-consanguineous single cases (n = 918) were 6-times as frequent as consanguineous cases (n = 151). In 595 patients with SRNS and mutations in recessive disease-causing genes the ascertainment of non-consanguineous single cases (n = 512) was also about 6-times as high as consanguineous cases (n = 83). Therefore, the ability to employ individuals from outbred populations in homozygosity mapping should greatly accelerate gene discovery.

Another strong limitation to gene identification is imposed by the experience that studies of consanguineous kindred result in segments of homozygosity that are too numerous and too large and contain too many positional candidates for efficient gene identification by mutation analysis. For instance, in our worldwide cohort individuals from consanguineous background had 4–60 cZLR peaks (Figure 3). These homozygous segments contained hundreds of positional candidate genes. Because of both limitations we wanted to evaluate whether gene identification by homozygosity mapping is possible in single individuals from outbred populations. Using a medium resolution SNP marker set (250 K) we were able to detect homozygous segments that contain the disease-causing mutations in single individuals from outbred populations as shown in Figure 3:

With the exception of 4 families (A1218, A646, F1158, and A825), all other 23 outbred families exhibited 4 or less cZLR peaks (Figure 3). In 2 of the 3 outbred families with 4 cZLR peaks, calculation of both affected individuals together reduced the number of cZLR peak from 4 and 3 cZLR peaks to 2 cZLR peaks (A237) and from 4 and 2 peaks to 1 cZLR peak (F60-61). Four outbred families had 3 cZLR peaks (F138, F456, F50, and F1183). Seven outbred individuals exhibited only 2 cZLR peaks (A762, F53, A1298, A567, A7, F54, and A1338) (Figure 3). In these individuals the cZLR peak that contained the homozygous gene mutation had a median size of 8.3 Mb (range 2.4 - 40.1 Mb) (Figure 3, insert).

Most interestingly, 4 outbred families exhibited only 1 cZLR peak (F399, A1730, F30_10G, and F408) (Figure 3). These peaks, which contained the homozygous gene mutation, had a median size of only 2.7 Mb (range 2.1–8.25). A genomic segment of 2.1 Mb is equivalent to an average of 16 candidate genes, a number of genes that permits gene identification by exon sequencing in candidate genes. For comparison, we have so far successfully identified disease genes by mutation analysis of all exons within intervals of 8.7 Mb (57 genes) in *NPHP5* [17], 1.5 Mb (9 genes) in *NPHP6* [18], 0.94 Mb (7 genes) in *NPHP3* [19], 0.90 Mb (11 genes) in *BSND* [20], 0.70 Mb (6 genes) in *NPHP4* [21], and 4.0 Mb (43 genes) in *PLCE1* [22]. We thereby demonstrated that we were able to detect a cZLR signal of “homozygosity by descent from the disease allele” above the background of “homozygosity by descent from haplotype blocks common to the outbred population”. To be detectable, the former would have to be more recent and therefore reside on a longer segment of homozygosity than the latter.

Finally, in 5 families (F409, A159, A1686, A1685, and A887), in whom the known homozygous mutation was positioned in homozygous segments of less than 2.1 Mb, we did not obtain any cZLR peak (see Table 1 and inset in Figure 3). Therefore, we conclude that the limit for detecting a homozygous segment in single outbred individuals with homozygous disease-causing mutations using this technique was 2.1 Mb. Three observations further substantiate this detection limit: i) We demonstrated for the first time that the R138Q mutation of *NPHS2* does in fact represent a founder mutation as it occurs on identical haplotypes (Figure S2). It was detected within a homozygous interval of 2.7 Mb (212 SNPs) in both individuals of family A1730 (Table 1 and Figure S2). It was also detected within 2.3 Mb of homozygosity in families A237, and A825, who had additional cZLR peaks on other chromosomes (Figure S2). However, the R138Q mutation was not detected within homozygous intervals of 1.2 Mb, 1.2 Mb, and 0.70 Mb, in families A159, A1686, and A887, respectively (Table 1 and inset in Figure 3). ii) In family F30 a single cZLR peak of 2.1 Mb width bearing a homozygous *NPHP4* mutation was detected in individual F30-1, but not within the sibling's (F30-2) interval of 1.14 Mb, which is shorter due to a recombination in the mother's meiosis (Table 1). We documented that the parents of F30 are related 10 generations ago. This gives an example of the “remoteness” of inbreeding that leads to homozygous intervals in the order of ∼1–2 Mb [23]. iii) Families F408 and F409 bear the same homozygous mutation (F142fsX14) in *NPHP5* and originate from the same geographic region in Switzerland. We demonstrate that this mutation is embedded in a shared haplotype (Figure S3). Whereas we detected the 5.18 Mb homozygous interval as a single cZLR peak in F408, the shorter haplotype of 0.65 Mb in F409 was not detected as a cZLR peak (Table 1 and Figure S3). In 28 unrelated individuals with recessive disease from outbred background with known homozygous mutations in recessive genes we failed to detect a “ZLR peak of homozygosity” in 6 (21%). In all of these patients the homozygous mutation resided in a homozygous segment of less than 2.1 Mb (Figure 3 and Table 1). Thus, the limit of detection of a homozygous mutation using our homozygosity mapping strategy was at 2.1 Mb (Table 1, Figures S2 and S3). It is remarkable however, that even if homozygous mutations were not detected as a cZLR peak they were always embedded in a homozygous haplotype of >0.65 Mb. This indicates that it may be possible to further improve the sensitivity of the approach.

**Table 1:** Detection limit of homozygosity by descent using 250 K SNP array.

Individual with known homozygous mutation	Gene with homozygous mutation	Number of cZLR peaks detected	Width of cZLR peak [Mb]	Number of homozygous SNPs
F399-1	*NPHP5*	1	*8.25	767
A1730-1	*NPHS2*	1	*2.70	212
A1730-2	*NPHS2*	1	*2.70	212
F30-1	*NPHP4*	1	*2.10	222
F30-2	*NPHP4*	0	1.14	125
F408	*NPHP5*	1	*5.18	420
F409	*NPHP5*	0	0.65	48
A159	*NPHS2*	0	1.20	108
A1686	*NPHS2*	0	1.20	89
A1685-1	*NPHP8*	0	0.70	49
A1685-2	*NPHP8*	0	0.70	49
A887	*NPHS2*	0	0.68	47
F190	*nc*	0	NA	NA

For individuals, in which total genome search yielded a single cZLR peak (homozygosity peak) that contains the homozygous disease-causing mutation, the width of the cZLR peak is marked with an asterisk. Note that the detection limit for homozygosity by descent was at 2.10 Mb.

cZLR, consistent likelihood ratio z-score (see Figure S1); NA, not applicable; nc, negative control individual with a known compound heterozygous mutation.

### False Positive cZLR Peaks Are Rare

The sensitivity for detecting a cZLR peak in an individual with a homozygous mutation was 93% (67/72) for all 72 patients (see above). It was 76% (16/21) if only the 21 outbred individuals with 4 or less cZRL peaks were evaluated. In order to assess the specificity of the method, i.e. whether or not cZLR peaks occur frequently as “false positives” in individuals without homozygous mutations in outbred populations, we examined 20 parents of affected individuals with a homozygous mutation, as parents are obligate heterogygotes. Thirteen of 20 such parents had no cZLR peak, equivalent to a specificity of 65%. Five parents had one cZLR peak, one parent had 2 peaks, and one father had 7 peaks, being most likely from unknown consanguineous background himself (Figure 3). This specificity of 65% should not pose a problem for gene identification, since the likelihood that a false posititive cZLR peak of a few Mb size will colocalize by chance for two individuals, thereby pointing to a false candidate locus, is in the order of only 1∶1,000.

In order to assess how likely it is to find a disease-causing mutation in outbred single affecteds with mutations in a recessive disease gene using the approach described here, we prospectively performed 250 k SNP DNA microarray analysis in 24 unrelated outbred patients with infantile nephrotic syndrome, which is known to be caused by *NPHS1* mutations in 22.5% of cases (18/80) [3] and by *PLCE1* mutations in 28% of cases (10/35) [24]. We obtained the following distribution for the number of ZLR peaks of homozygosity per patient: 0, 1, 2, 4, and 6 peaks in 9, 4, 8, 1, and 2 patients, respectively. In all 24 patients mutation analysis was performed by exon PCR of all *NPHS1* and *PLCE1* exons. We identified both causative mutations in 21% (5/24) of these patients (4 patients had *NPHS1* mutations and 1 patient had a *PLCE1* mutation). In the 15 of 24 outbred patients that had ZLR peaks of homozygosity, we identified homozygous mutations in 27% (4/15) of these patients (3 patients with a homozygous *NPHS1* mutation and 1 patient with a homozygous *PLCE1* mutation). As expected, these 4 patients with homozygous mutations had a ZLR peak of homozygosity at the locus of their mutation. However 1 additional patient had a compound heterozygous mutation of *NPHS1* although we observed 2 ZLR peaks of homozygosity at different loci. This patient represents an example of a “false positive result” in the homozygosity mapping approach. We conclude that the fraction of homozygous mutations detected in *NPHS1* and *PLCE1* in patients with infantile nephrotic syndrome (27%) using the homozygosity mapping approach in outbred individuals prospectively was similar to the one reported by diagnostic sequencing (22.5% for *NPHS1* and 28% for *PLCE1*).

We conclude that homozygosity mapping of recessive disease genes is possible in single individuals from outbred populations. The current resolution is at ∼2.1 Mb, containing an average of only 16 candidate genes.

## Discussion

In a worldwide cohort of 72 individuals from 54 families with homozygous mutations of 13 different autosomal recessive disease genes we here describe a strategy for the detection of disease loci by homozgosity mapping using 250 K SNP arrays. The number of cZLR peaks reflected the degree of inbreeding and thereby the extent of homozygosity by descent. We were able to detect the locus of the disease-causing mutation in 93% of cases. We demonstrate for the first time in a systematic way that loci of homozygous mutations in recessive disease-causing genes can be detected in single individuals from outbred populations. The detection limit was at 2.1 Mb of a segment for homozygosity by descent.

Recently, data was published on segments of homozygosity by descent (“runs of homozygosity, ROH”) from inbred and outbred populations [25]. The outbred population studied by McQuillan et al. that most closely resembles our outbred population is the “HapMap CEU” population. The CEU population is a Northwest-European-derived population from Utah, USA, which represents the founders of the CEPH individuals (Centre d'Etude du Polymorphisme Humain), in which broad genetic studies have been performed for decades. Interestingly, ROH of <2.5 Mb size were detected in close to 100% of CEU individuals, whereas ROH of 2.5–5.9 Mb were found in only 22%, and ROH for 5–9.9 Mb in only 5% of individuals. These data compare with our study as follows: Using the calling method for “ZLR peaks of homozygosity” as described in our study, we did not detect any peaks if a known mutation resided in a homozygous segment of less than 2.7 Mb (with one exception of a peak detected at 2.1 Mb) (see Table 1). This implies that the high background of ROH<2.5 Mb seen in the study by McQuillan et al. in outbred individuals would not pose a problem to our approach. Our study would rather have to be compared (in randomly selected outbred individuals) to a rate of background “ZLR peaks of homozygosity” of 22% for 2.5–5.9 Mb ROH and with 5% for 5–9.9 Mb ROH found in the McQuillan study. In our study, for 18 individuals that self-described as “non-consanguineous” and exhibited “ZLR peaks of homozygosity”, 11 of them (61%) had 1 or 2 “ZLR peaks” spanning 2.1–11.5 Mb of total genomic width (Figure 3). Thus, we found a very large enrichment of ROH in individuals with a known homozygous mutation in a recessive gene compared to randomly-selected healthy control subjects.

If feasibility of gene identification is considered in this setting, a span of genomic sequence of 12 Mb would harbor on average less than 1,800 exons (12 Mb×6 genes/1 Mb×25 exons per gene). Using new exon capture techniques followed by large-scale sequencing [26] it will be feasible to capture candidate regions from more than 13 such individuals on a single DNA capture microarray using the current technology that allows capture and sequencing of a total of 5 Mb of sequence on one DNA capture microarray (13 individuals×1,800 exons×200 bp/exon = 4.68 Mb).

### The Utility of the Approach

These data are, to our knowledge, the first to quantify for a large cohort of patients with known homozygous mutations in a high number of different recessive disease-causing genes the extent of homozygosity by descent and its detection limit in outbred populations. Our findings have the potential to strongly facilitate the identification of recessive disease-causing genes by overcoming some of its major impediments: i) First, large cohorts of consanguineous pedigrees are very difficult to ascertain, whereas most pediatric specialty clinics have direct access to sufficient numbers of single individuals of rare pediatric diseases. ii) Second, homozygosity mapping in consanguineous kindred leads to large candidate regions that usually contain too many positional candidate genes to allow for gene identification by mutation analysis. Here we demonstrate that homozygosity mapping can be performed in single affecteds from outbred populations yielding a single cZLR peak per genome. We were able to map the disease-causing gene to single peaks representing segments of homozygosity that were as short as 2.1 Mb, which would contain 16 genes on average. For positional cloning of novel recessive disease genes this strategy offers a reduction of complexity of three orders of magnitude, i.e. from 3,300 Mb to ∼3.3 Mb or from 25,000 genes to ∼25 genes. This reduction makes gene discovery by mutation analysis feasible, either by exon sequencing or large-scale sequencing using emerging techniques.

Additionally, in regions of homozygosity affected individuals from different families that originate from the same geographic region may, within the homozygous haplotype, be identical by descent from an unknown common ancestor. Candidate gene analysis can then be focused on this short interval under the hypothesis that both families might share the same disease allele, as we show for the *NPHS2* mutation R138Q (Figure S2) and for an *NPHP5* founder mutation (Figure S3). This approach has also been useful in the identification of the *NPHP6/CEP290* gene [18].

### Application to Molecular Genetic Diagnostics

The strategy described here is especially useful in situations of pronounced genetic locus heterogeneity, in which similar autosomal recessive phenotypes may be caused by a high number of different genes. In this situation a first run of homozygosity mapping will show whether the individual most likely carries a homozygous disease mutation by presence of one or more cZLR peaks. Mutation analysis by exon sequencing can then be restricted to genes that are positioned in a cZLR peak of the individual. An example for NPHP is given in Figure 2B. Even if in the near future rapid and cost-effective sequencing of a person's genome will be possible, which has been referred to as the “$1,000 genome”, homozygosity mapping in outbred populations will be a valuable first step of molecular genetic diagnostics in recessive diseases for the following reason: It generates the hypothesis that a disease-causing homozygous mutation will be found in a homozygosity peak and nowhere else, thereby reducing the high number of difficult-to-interpret sequence changes found outside cZLR peaks.

### Improving the Detection Rate

Our data show that the approach of homozygosity mapping in outbred individuals at a marker density of 250 K has not reached its theoretical limit of refinement yet, as we found some homozygous disease alleles embedded in haplotypes as short as 0.65 Mb equivalent to only 48 SNP of the 250 K array (Table 1, Figures S2 and S3). The following measures may increase the likelihood of detecting even shorter unique homozygous segments that contain the disease-causing mutation in single individuals from outbred populations: i) When increasing the density of markers evaluated in multipoint calculations to more than 1 marker per 100,000 nucleotides, additional homozygosity peaks appear as “background”. This background is most likely due to non-informative alleles (identically homozygous in both parents). Linkage calculation together with parental genotypes will allow exclusion of these markers, thereby reducing the number of false positive cZLR peaks. This will permit increasing the number of markers evaluated, allowing detection of even smaller homozygous loci. iii) SNP arrays with higher density are now available (e.g. 1 Mill. SNPs). They contain HapMap-derived SNPs that are potentially more informative. However, as we observed a relatively high rate (<4%) of false heterozygous allele calls in the 250 K SNP arrays (see Methods), the utility of higher density SNPs for homozygosity mapping in outbred individuals will need to be tested first. The individuals tested here, in whom we showed that homozygous mutations are embedded in a short homozygous haplotype by descent, provide a perfect resource for testing the limitations of higher resolution SNP arrays for homozygosity mapping in outbred individuals. As inclusion of parents of an affected individual in the total genome haplotype analysis establishes the origin of the paternal and maternal haplotypes, a data base of disease-allele specific haplotypes would permit even detection of known mutations by their haplotype in individuals with compound heterozygous mutations in the future.

The new strategy of homozygosity mapping in single outbred individuals described here has a strong potential of accelerating the discovery of novel recessive disease genes as a critical step towards elucidating the pathogenesis of a wide variety of pediatric disorders independent of organ system involved.

## Supporting Information

Figure S1 Homozygosity profiles reveal loci of homozygous mutations in recessive disease genes.
(11.18 MB TIF)

Figure S2 The European “founder” mutation R138Q of NPHS2 occurs on a shared haplotype by descent from a common ancestor.
(4.30 MB PDF)

Figure S3 The NPHP5 mutation F142fsX147 occurs on a shared haplotype.
(2.48 MB PDF)

Table S1 Genetic characteristics of 72 individuals from 54 unrelated families with NPHP or SRNS.
(0.23 MB DOC)
