# Supplementary material for: A Systematic Approach to Mapping Recessive Disease Genes in Individuals from Outbred Populations
Source: PLoS Genet. 2009 Jan 23;5(1):e1000353. doi: 10.1371/journal.pgen.1000353 (PMC2621355; doi:10.1371/journal.pgen.1000353)
Supplement: Figure S2 — The European “founder” mutation R138Q of NPHS2 occurs on a shared haplotype by descent from a common ancestor. (4.30 MB PDF) [file pgen.1000353.s002.pdf]

Chromosome	Physical.Position	dbSNP.RS.ID	A1730 II-1	A1730 II-2	A825 II-1	A237 II-1	A237 II-2	A646 II-1	A1686 II-2	A159 II-1	A887 II-1
1	174541217	rs10913445	AB	AB	AB	AB	AB	AB	AB	AB	BB
1	174541284	rs10798574	AB	NoCall	AB	AB	AB	BB	AB	AB	AB
1	174541518	rs10913446	AA	AA	AB	AB	AB	AA	AB	AB	AB
1	174542963	rs12143390	AA	AA	AA	AA	AA	AA	AA	AA	AB
1	174543012	rs7547105	BB	BB	BB	BB	BB	BB	BB	BB	BB
1	174543156		BB	BB	BB	BB	BB	BB	BB	BB	AB
1	174543674	rs3886720	BB	BB	BB	BB	BB	BB	BB	BB	AB
1	174547426	rs12757638	AA	AA	AB	AB	AB	AA	AB	AB	BB
1	174560544	rs16852012	BB	BB	BB	BB	BB	BB	BB	BB	BB
1	174584446	rs10913454	BB	BB	AB	BB	BB	BB	BB	AB	AB
1	174584877	rs10798577	NoCall	BB	AB	BB	BB	BB	BB	AB	AB
1	174613829	rs2039403	BB	BB	BB	AB	AB	BB	BB	BB	AB
1	174623983	rs2282287	AA	AA	AA	AB	AB	AA	AA	AA	AB
1	174630456	rs623479	AA	AA	AA	AA	AA	AA	AA	AA	AB
1	174631219	rs626976	AA	AA	AA	AA	AA	AA	AA	AA	AB
1	174634139	rs3813648	AA	AA	AB	AA	AA	AA	AA	AB	AA
1	174636152	rs10489882	BB	BB	AB	AB	AB	BB	BB	AA	AB
1	174636628	rs10913465	BB	BB	BB	BB	BB	BB	BB	BB	AB
1	174643864	rs4652279	BB	BB	BB	BB	BB	BB	AB	BB	AB
1	174647131	rs842192	BB	BB	BB	BB	BB	BB	AB	AB	BB
1	174661597	rs7413442	AA	AA	AA	AA	AA	AA	AA	AA	AA
1	174666707	rs1889981	BB	BB	AB	AB	AB	BB	AB	AB	AA
1	174666860	rs954502	BB	BB	AB	AB	AB	BB	BB	BB	AA
1	174666932	rs1415085	AA	AA	AA	AA	AA	AA	AA	AA	AA
1	174675469	rs869286	AA	AA	AB	AB	AB	AA	AB	BB	BB
1	174681883	rs1556975	AA	AA	AB	AB	AB	AA	AB	BB	BB
1	174695228	rs10913483	BB	BB	AB	AB	AB	BB	AB	AA	AA
1	174699854	rs1336779	BB	BB	BB	BB	BB	BB	AB	AA	AA
1	174700896	rs16852325	AA	AA	AB	AB	AB	AA	AA	AA	AA
1	174700972	rs4652286	BB	BB	BB	BB	BB	BB	AB	AB	AB
1	174701023	rs4652287	AA	AA	AA	AA	AA	AA	AB	BB	BB
1	174702804	rs12409286	AA	AA	AB	AB	AB	AA	AA	AB	AB
1	174709363	rs12078421	AA	AA	AA	AA	AA	AA	AA	AA	AA
1	174711465	rs1932847	BB	BB	BB	BB	BB	BB	AB	AB	AB
1	174711877	rs7531837	AA	AA	AA	AA	AA	AA	AB	AB	AB
1	174726129	rs946819	BB	BB	BB	BB	BB	BB	BB	AB	AB
1	174740130	rs10753177	AA	AA	AA	AA	AA	AA	AA	AB	AB
1	174757951	rs7520853	BB	BB	BB	BB	BB	BB	BB	BB	AB
1	174762258	rs16852397	AA	AA	AA	AA	AA	AA	AA	AA	AA
1	174767512	rs6681729	BB	BB	BB	BB	BB	BB	BB	BB	BB
1	174827461	rs12071903	AA	AA	AA	AA	AA	AA	AA	AA	AA
1	174873853	rs10047059	BB	BB	BB	BB	BB	BB	BB	BB	BB
1	174911914	rs963607	AA	AA	AA	AA	AA	AA	AA	AA	AA
1	174912376	rs10798600	AA	AA	AA	AA	AA	AA	AA	AA	AA
1	174938285	rs10913522	BB	BB	BB	BB	BB	BB	BB	BB	BB
1	175001321	rs10913531	BB	BB	BB	BB	BB	BB	BB	BB	BB
1	175040568	rs11805590	AA	AA	AA	AA	AA	AA	AA	AA	AA
1	175078428	rs1510266	BB	BB	BB	BB	BB	BB	BB	BB	BB
1	175105774	rs7418912	AA	AA	AA	AA	AA	AB	AA	AA	AB
1	175109329	rs16852787	AA	AA	AA	AA	AA	AA	AA	AA	AA
1	175120379	rs2902237	AA	AA	AA	AA	AA	AB	AA	AA	AB
1	175181961	rs6693257	BB	BB	BB	BB	BB	BB	BB	BB	BB
1	175184286	rs10913560	AA	AA	AA	AA	AA	AA	AA	AA	AA
1	175189519	rs1397187	BB	BB	BB	BB	BB	BB	BB	BB	BB
1	175190365	rs1008495	AA	AA	AA	AA	AA	AA	AA	AA	AA
1	175198426	rs6666248	AA	AA	AA	AA	AA	AB	AA	AA	AB
1	175202522	rs10158298	AA	AA	AA	AA	AA	AA	AA	AA	AA
1	175214539	rs12096233	AA	AA	AA	AA	AA	AA	AA	AA	AA
1	175214641	rs10798610	AA	AA	AA	AA	AA	AB	AA	AB	AB
1	175227898	rs6668819	AA	AA	AA	AA	AA	AB	AA	AA	AA
1	175240587	rs6700266	BB	BB	BB	BB	BB	AB	BB	BB	BB
1	175246969	rs4650994	AA	AA	AA	AA	AA	AB	AA	AA	AA
1	175252540	rs4075703	NoCall	AB	AA	AA	AA	AA	AA	AA	AA
1	175263845	rs4650999	AA	AA	AA	AA	AA	AB	AA	AA	AA
1	175263880	rs4651000	BB	BB	BB	BB	BB	AB	BB	AB	AB
1	175283102	rs6685710	BB	BB	BB	BB	BB	BB	BB	BB	BB
1	175283129	rs2811297	AA	AA	AA	AA	AA	AB	AA	AA	AA
1	175284532	rs2811302	AA	AA	AA	AA	AA	AB	AA	AA	AA
1	175291816	rs2811271	BB	BB	BB	BB	BB	AB	BB	AB	AB
1	175305874	rs2811291	AA	AA	AA	AA	AA	AB	AA	AB	AB
1	175315506	rs2761467	AA	AA	AA	AA	AA	AA	AA	AA	AA
1	175320711	rs7545103	AA	AA	AA	AA	AA	AB	AA	AA	AA
1	175350840	rs2811314	AA	AA	AA	AA	AA	AB	AA	AB	AB
1	175351557	rs2476561	BB	BB	BB	BB	BB	AB	BB	AB	AB
1	175352753	rs10913590	BB	BB	BB	BB	BB	BB	BB	BB	BB
1	175357156	rs2761476	AA	AA	AA	AA	AA	AA	AA	AB	AB
1	175357677	rs6690886	AA	AA	AA	AA	AA	AA	AA	AB	AB
1	175360668	rs10798624	BB	BB	BB	BB	BB	BB	BB	AB	AB
1	175372254	rs10753183	BB	BB	BB	BB	BB	BB	BB	BB	BB
1	175377217	rs10913603	BB	BB	BB	BB	BB	BB	BB	BB	BB
1	175388622	rs2811248	AA	AA	AA	AA	AA	AA	AA	AA	AA
1	175388695	rs12407833	AA	AA	AA	AA	AA	AA	AA	AA	AA
1	175389060	rs2773090	AA	AA	AA	AA	AA	AA	AA	AA	AB
1	175393797	rs2811261	BB	BB	BB	BB	BB	BB	BB	BB	AB
1	175400769	rs2773098	AA	AA	AA	AA	AA	AA	AA	AA	AB
1	175423023	rs10913619	BB	BB	BB	BB	BB	BB	AB	BB	AB
1	175441623	rs4652319	AA	AA	AA	AA	AA	AA	AB	AA	AB
1	175477268	rs6662050	BB	BB	BB	BB	AB	BB	BB	BB	BB
1	175569656	rs17725459	AA	AA	AA	AA	AA	AA	AA	AA	AA
1	175651683	rs12138385	AA	AA	AA	AA	AA	AA	AA	AA	AA
1	175655082	rs2039532	AA	AA	AA	AA	AA	AA	AA	AB	BB
1	175677624	rs10913665	BB	BB	BB	BB	BB	AB	AB	AB	AB
1	175721121	rs7520456	BB	BB	BB	BB	BB	AB	BB	AB	BB
1	175765886	rs12131503	AA	AA	AA	AA	AA	AA	AA	AA	AA
1	175769812	rs12083856	AA	AA	AA	AA	AA	AA	AA	AA	AA
1	175777618	rs3766623	AA	AA	AA	AA	AA	AA	AA	AA	AA
1	175780080	rs10798651	BB	BB	BB	BB	BB	AB	BB	AB	BB
1	175782135	rs3813639	AA	AA	AA	AA	AA	AA	AA	AA	AA
1	175784708	rs4652355	BB	BB	BB	BB	BB	BB	BB	BB	BB
1	175792765	rs1570807	AA	AA	AA	AA	AA	AA	AA	AA	AA
1	175793084	rs10798652	AA	AA	AA	AA	AA	AA	AA	AA	AA
1	175798728	rs10913690	BB	BB	BB	BB	BB	BB	BB	BB	BB
1	175835358	rs2636287	BB	BB	BB	BB	BB	BB	BB	BB	BB
1	175933866	rs2793799	BB	BB	BB	BB	BB	BB	BB	BB	BB
1	175942333	rs2816169	BB	BB	BB	BB	BB	BB	BB	BB	BB
1	175955383	rs1078872	BB	BB	BB	BB	BB	BB	BB	BB	BB
1	175966135	rs425858	BB	BB	BB	BB	BB	BB	BB	BB	BB
1	175984249	rs16853837	BB	BB	BB	BB	BB	BB	BB	BB	BB
1	176015297	rs4652363	AA	AA	AA	AA	AA	AA	AA	AA	AB
1	176083994	rs4652367	BB	BB	BB	BB	BB	BB	BB	BB	BB
1	176131917	rs10913753	BB	BB	BB	BB	BB	BB	BB	BB	BB
1	176135670	rs6659294	AA	AA	AA	AA	AA	AA	AA	AA	AA
1	176139065	rs6683623	AA	AA	AA	AA	AA	AA	AA	AA	AA
1	176143754	rs6664339	AA	AA	AA	AA	AA	AA	AA	AA	AA
1	176158047	rs12740455	AA	AA	AA	AA	AA	AA	AA	AA	AA
1	176158499	rs10913776	BB	BB	BB	BB	BB	BB	BB	BB	BB
1	176183932	rs10494520	AA	AA	AA	AB	AA	AA	AA	AA	AA
1	176190305	rs10913797	AA	AA	AA	AA	AA	AA	AA	AA	AA
1	176191211	rs6674086	BB	BB	BB	BB	BB	BB	BB	BB	BB
1	176191333	rs6674274	AA	AA	AA	AA	AA	AA	AA	AA	AA
1	176218255	rs7512551	BB	BB	BB	BB	BB	BB	BB	BB	BB
1	176228436	rs11805319	BB	BB	BB	BB	BB	BB	BB	BB	BB
1	176228630	rs964982	BB	BB	BB	BB	BB	BB	BB	BB	BB
1	176228676	rs964983	AA	AA	AA	AA	AA	AA	AA	AA	AA
1	176228721	rs964984	AA	AA	AA	AA	AA	AA	AA	AA	AA

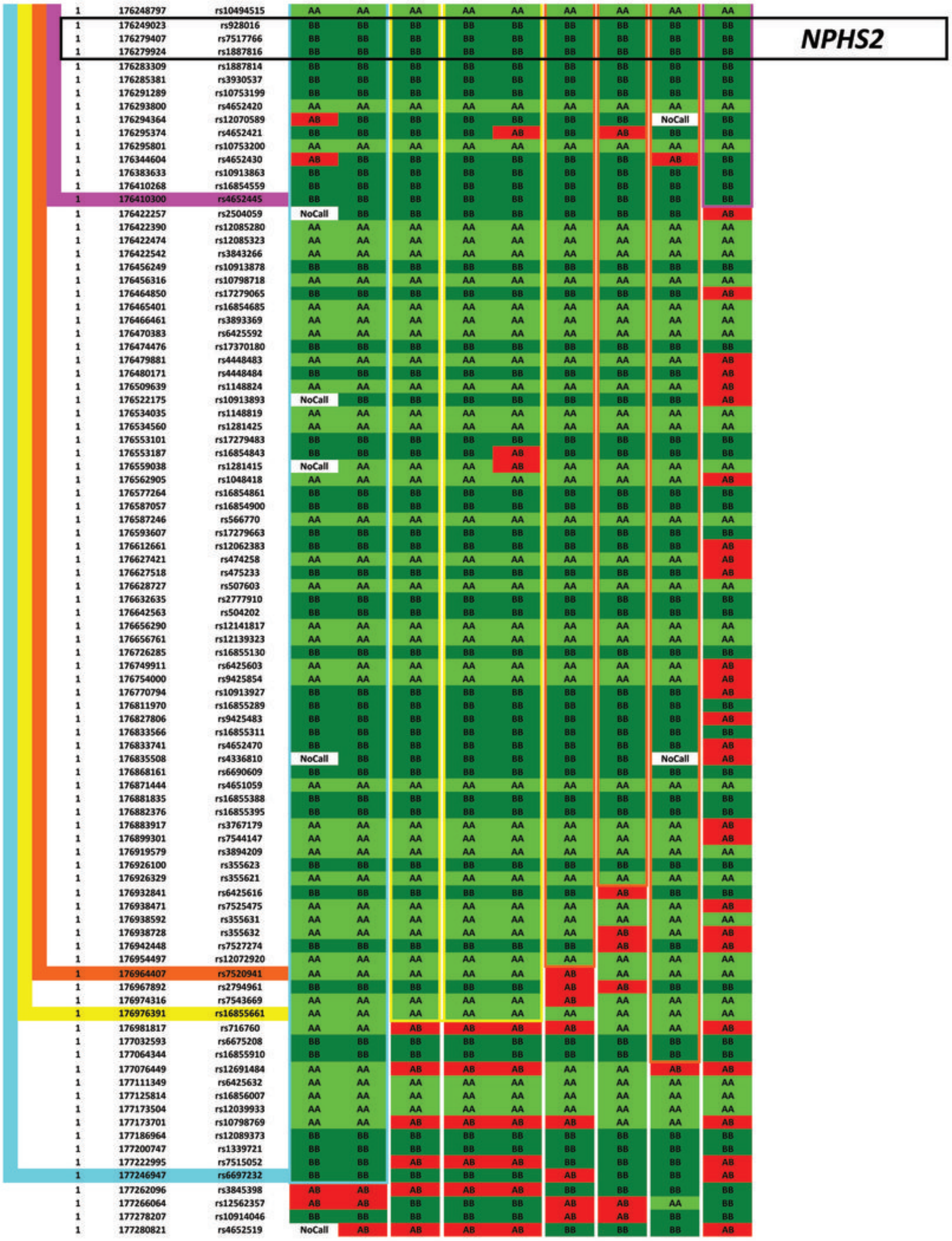

Supplementary Figure 2. The European "founder" mutation R138Q of *NPHS2* occurs on a shared haplotype by descent from a common ancestor.

Haplotypes from a 250k SNP array of 7 different families with steroid resistant nephrotic syndrome due to the homozygous European "founder" mutation R138Q of *NPHS2* are shown. Homozygous alleles are on green background (light green for "AA", dark green for "BB"). Heterozygous alleles are on red background. "No calls" by the SNP evaluation software BRLMM are on white background. Continous segments of homozygosity (that are partially interrupted by rare false heterozygous allele calls) are encased in colored boxes and delimited by same-color brackets on the left with their heterozygous flanking markers indicated in the same color. The position of the *NPHS2* gene is indicated.

Note that all families within the homozygous region share identical homozygous alleles (with the exception of a few false heterozygous allele calls). In this way we demonstrate that the European "founder" mutation R138Q of *NPHS2* does in fact occur by descent from a founder as proposed (Boute et al. 2000). The homozygous intervals measure 2.70 Mb in A1730, 2.3 Mb in A825 and A237 ~1.2 Mb in A646, A1686 and A159, and 0.68 Mb in A887. Whereas the intervals of 2.70 Mb were detectable as a cZLR peak the shorter intervals were not (see **Table 1** and **Figure 3**).
