# Supplementary material for: A Systematic Approach to Mapping Recessive Disease Genes in Individuals from Outbred Populations
Source: PLoS Genet. 2009 Jan 23;5(1):e1000353. doi: 10.1371/journal.pgen.1000353 (PMC2621355; doi:10.1371/journal.pgen.1000353)
Supplement: Figure S3 — The NPHP5 mutation F142fsX147 occurs on a shared haplotype. (2.48 MB PDF) [file pgen.1000353.s003.pdf]

Chromosom	Physical.Po	dbSNP.RS.I	F1	F399	A567	F408	F409
3	108,312,022	rs1919008	AB	AA	AA	AA	AA
3	108,333,111	rs7640923	AB	BB	BB	NoCall	BB
3	108,450,567	rs1283098	BB	AA	AB	AA	BB
3	108,457,213	rs793616	AA	AA	AA	AA	AA
3	108,458,089	rs1298166	AA	BB	NoCall	AB	AA
3	108,458,589	rs793612	AA	AA	AA	AA	AA
3	108,471,129	rs9867626	BB	AB	BB	BB	BB
3	108,471,743	rs3909653	AA	AA	AA	AA	AA
3	108,523,998	rs10933897	AA	BB	AB	AB	AA
3	108,556,931	rs709544	BB	BB	BB	BB	BB
3	108,557,174	rs709545	AA	AB	AA	AA	AA
3	117,586,473	rs16824826	AA	AA	AA	AA	AA
3	117,587,211	rs17647091	NoCall	AB	AB	AA	AB
3	117,604,638	rs7637573	AA	AA	AB	AB	AB
3	117,604,662	rs9877364	BB	BB	BB	BB	BB
3	117,613,898	rs1731596	AA	AA	AB	AB	AB
3	117,643,669	rs16824908	AA	AB	AB	AB	AB
3	117,666,398	rs1712961	BB	AB	BB	BB	BB
3	117,667,176	rs6787168	AA	AA	AB	AB	AA
3	117,686,991	rs4530520	NoCall	AB	AA	AA	AA
3	117,712,301	rs9810534	BB	BB	BB	BB	BB
3	117,712,345	rs10934349	AA	BB	AB	AB	AB
3	117,728,759	rs17706517	AA	BB	BB	BB	AB
3	117,755,493	rs9851626	BB	BB	BB	BB	BB
3	117,759,851	rs10451890	BB	AA	AA	AA	AB
3	117,767,323	rs1795287	AA	NoCall	AB	AB	AA
3	117,767,630	rs1795285	BB	AA	AA	AA	AB
3	117,768,487	rs1712983	BB	BB	AB	AB	BB
3	117,771,489	rs1712987	NoCall	AA	AB	AB	AA
3	117,796,334	rs1518325	AA	AA	AB	AB	AA
3	117,798,780	rs1356535	BB	BB	BB	BB	BB
3	117,869,549	rs1543176	AA	NoCall	AA	AA	AA
3	117,869,742	rs6795004	AA	AA	AA	AA	AA
3	117,890,039	rs16825309	BB	BB	AB	BB	BB
3	117,912,015	rs1518338	NoCall	BB	AB	BB	AB
3	117,914,112	rs1464208	BB	BB	BB	BB	NoCall
3	117,915,816	rs12488450	AA	AA	AB	AA	AA
3	117,926,410	rs1991744	BB	BB	AB	BB	AB
3	117,926,566		AA	AA	AA	AA	AA
3	117,927,395	rs1432363	NoCall	AA	AB	AA	AA
3	117,927,772	rs1347055	AA	AA	AA	AA	AB
3	117,928,014	rs1432364	AA	AA	AB	AA	AB
3	117,928,158	rs7618068	AA	AA	AB	AA	NoCall
3	117,928,191	rs2867841	NoCall	BB	AB	BB	AB
3	117,932,079	rs1946600	AA	AA	AA	AA	AA
3	117,932,789	rs1432373	AA	AA	AA	AA	AA
3	117,959,735	rs13077521	AA	AA	AA	AA	BB
3	117,963,549	rs1000357	AB	BB	BB	BB	BB
3	117,963,712	rs1000356	AA	AA	AA	AA	AA
3	117,963,741	rs1000355	AA	AA	NoCall	AB	BB
3	117,983,605	rs6788352	AA	AA	AA	AB	AB
3	117,984,074	rs17660641	AA	AA	BB	AA	AA
3	118,019,154	rs7627376	AA	BB	AB	BB	AA
3	118,019,250	rs7617143	AA	BB	AB	BB	AA
3	118,041,025	rs6438347	BB	BB	AB	BB	BB
3	118,056,506	rs7610948	BB	BB	BB	BB	BB
3	118,097,650	rs7614729	BB	BB	BB	BB	BB
3	118,107,684	rs1104956	NoCall	BB	BB	BB	BB
3	118,136,647	rs517452	AA	AA	AB	AB	BB
3	118,137,913	rs7651803	NoCall	BB	BB	BB	NoCall
3	118,141,555	rs3972692	AA	AA	AA	AB	AB
3	118,153,583	rs940161	AA	AA	AA	AA	AA
3	118,169,072	rs2177331	BB	BB	BB	BB	BB
3	118,193,155	rs1518902	AA	AA	AA	AA	AA
3	118,194,547	rs558329	AA	BB	AB	AA	AA
3	118,205,020	rs9846364	AA	BB	BB	AA	AB
3	118,208,105	rs6783180	BB	AA	AB	BB	BB
3	118,219,429	rs3860538	BB	BB	BB	BB	BB
3	118,219,685	rs2090587	AA	AA	BB	AA	AB
3	118,244,350	rs1521418	BB	BB	AA	BB	BB
3	118,267,542	rs9820102	AA	BB	AA	AB	AB
3	118,273,785	rs7652831	AA	AA	AA	AA	AA
3	118,280,357	rs11710602	AA	AA	AA	AA	AA
3	118,281,770	rs7611371	NoCall	AB	BB	BB	BB
3	118,281,795	rs1357102	BB	BB	BB	BB	BB
3	118,282,084	rs9842731	BB	AB	AA	AB	AB
3	118,285,001	rs10934383	BB	BB	BB	BB	AB
3	118,301,805	rs9866334	BB	BB	BB	BB	BB
3	118,306,583	rs1357100	AA	AA	AA	AA	AA
3	118,314,115	rs1474237	AA	AA	AB	AA	AA
3	118,334,584	rs13098400	AA	AA	AA	AB	AA
3	118,335,159	rs6808814	AA	AA	AA	AB	AA
3	118,341,685	rs9289074	AA	AA	AA	AB	AA
3	118,345,185	rs1462840	BB	BB	BB	AB	BB
3	118,375,759	rs6766837	AA	AA	AB	AB	AA
3	118,377,112	rs6770523	BB	BB	BB	BB	BB
3	118,399,290	rs13061678	BB	BB	AB	AB	AB
3	118,404,421	rs17698379	BB	BB	BB	BB	BB
3	118,425,700	rs1462845	AA	AA	AA	AB	AB
3	118,444,327	rs1462850	BB	NoCall	AB	BB	BB
3	118,445,897	rs2126599	BB	BB	BB	BB	BB
3	118,453,893	rs6775136	BB	BB	BB	AB	AB
3	118,455,010	rs6791683	AA	AA	AA	AA	AA
3	118,455,161	rs16826476	NoCall	AA	AA	AA	AA
3	118,496,755	rs4855941	BB	AA	AA	AA	AA
3	118,538,955	rs1355711	AA	AA	AA	AA	AA
3	118,543,674	rs13074405	BB	AA	AB	AA	AA
3	118,559,909	rs1398641	AA	AA	AB	BB	BB
3	118,560,026	rs1398640	AA	AA	AA	AA	AA
3	118,564,666	rs6793644	AA	AA	AA	AA	AA
3	118,566,641	rs6438390	BB	BB	AB	AA	AA
3	118,567,115	rs2869782	BB	BB	BB	BB	BB
3	118,578,031	rs2048610	AA	AA	AB	BB	BB
3	118,582,861	rs9842624	AA	NoCall	AA	AA	AA
3	118,583,257	rs1513160	AA	AA	AA	AA	AA
3	118,584,248	rs16826761	AA	AA	AA	AA	AA
3	118,610,260	rs13326504	AA	AA	AA	AB	AB
3	118,618,766	rs1513161	AA	AA	AB	BB	BB
3	118,631,671	rs6800337	BB	BB	BB	BB	BB
3	118,675,868	rs1354152	AA	BB	BB	BB	AB
3	118,680,717	rs1676231	BB	AA	AA	AA	AB
3	118,680,875	rs1676230	AA	BB	BB	BB	BB
3	118,682,441	rs1698041	AA	BB	BB	BB	AB
3	118,716,100	rs2937671	AA	AA	AA	AA	AA
3	118,717,529	rs1676232	AA	BB	BB	BB	AB
3	118,725,726	rs16827097	AA	AA	AA	AA	AA
3	118,726,699		AA	AA	AA	AA	AA
3	118,727,194	rs9861389	AA	AA	AA	AA	AA
3	118,767,519	rs7620877	BB	AA	AB	BB	BB
3	118,775,438	rs1903736	BB	BB	BB	BB	BB
3	118,775,668	rs9867372	BB	AA	AB	BB	BB
3	118,812,945	rs1354147	BB	AA	AB	BB	AB
3	118,813,406	rs1501871	AA	BB	AB	AA	AB
3	118,813,548	rs1847023	BB	AA	AB	BB	AB
3	118,818,111	rs1354151	BB	AA	AB	BB	AB
3	118,826,690	rs4146331	BB	AA	AA	BB	AB
3	118,834,895	rs10934409	AA	BB	AA	AA	AA
3	118,838,929	rs6804100	AB	BB	AA	NoCall	AA
3	118,857,458	rs4404477	BB	AA	BB	BB	BB
3	118,877,663	rs9810976	AA	BB	AA	AA	AA
3	118,878,394	rs4549308	AA	BB	AA	AA	AA
3	118,893,188	rs3923050	AA	BB	AA	AA	AA
3	118,957,147	rs4602427	AA	AA	BB	BB	BB
3	118,957,425	rs9858155	BB	BB	BB	BB	BB
3	118,965,377	rs4687858	BB	BB	AA	AA	AA
3	118,972,549	rs4571264	AA	AA	AA	AA	AA
3	118,976,290	rs4146336	AA	AA	NoCall	AA	AA
3	118,989,279	rs11926918	BB	NoCall	AB	AA	AB
3	118,999,434	rs6786565	AA	BB	AB	BB	AB
3	119,002,987	rs6799997	NoCall	NoCall	AA	AA	AA
3	119,032,075	rs7652546	AB	AA	AA	AA	AA
3	119,064,112	rs4688066	AA	AA	AA	AA	AA
3	119,127,487	rs7645813	BB	BB	BB	BB	BB
3	119,129,604	rs4146374	AA	AA	AA	AA	AA
3	119,133,607	rs1350160	BB	BB	BB	BB	BB
3	119,143,663	rs1350158	AA	AA	BB	BB	BB
3	119,173,668	rs9849010	AA	BB	AA	AA	AA
3	119,181,490	rs2605532	AA	AA	AB	AA	AA
3	119,181,658		BB	BB	BB	BB	AB
3	119,181,729	rs1499968	BB	BB	AB	BB	BB
3	119,239,041	rs10934431	BB	BB	BB	BB	AB
3	119,239,225	rs843855	AA	AA	AB	AA	AA
3	119,256,614	rs10212130	AA	BB	AB	AA	AA
3	119,283,235	rs1392265	AA	BB	AB	AA	AA
3	119,284,020	rs1499976	BB	AA	NoCall	BB	NoCall
3	119,288,028	rs4399884	AA	BB	AB	AA	AA
3	119,299,896	rs17724734	AA	AA	AA	AA	AB
3	119,308,208	rs6794842	BB	AA	BB	BB	BB
3	119,314,205	rs4688078	AA	BB	AB	AA	AB
3	119,331,709	rs1486337	AA	BB	AA	AA	AB
3	119,374,423	rs6438441	BB	AA	BB	BB	BB
3	119,376,519	rs9857311	BB	BB	BB	BB	BB
3	119,392,442	rs2865313	BB	BB	BB	BB	BB
3	119,409,587	rs6775526	BB	AA	AA	BB	AB
3	119,414,890	rs4490352	AA	BB	BB	AA	AA
3	119,416,638	rs4493424	AA	BB	BB	AA	AA
3	119,446,756	rs12695364	BB	AA	AA	BB	BB
3	119,480,070	rs1696532	AA	BB	BB	AA	AA
3	119,480,584	rs7639542	AA	AA	AA	AA	AA
3	119,481,471	rs11719223	AA	AA	BB	AA	AA
3	119,483,167	rs16828176	BB	BB	BB	BB	BB
3	119,503,622	rs705260	BB	BB	AA	BB	BB
3	119,513,892	rs779596	BB	BB	AA	BB	BB
3	119,515,366	rs1083759	AA	BB	AA	AA	AA
3	119,543,880	rs16828239	BB	BB	BB	BB	BB
3	119,548,789	rs1348969	AA	AA	BB	AA	AA
3	119,556,561	rs7372488	AA	AA	BB	AA	AA
3	119,556,585	rs3906931	BB	BB	BB	NoCall	BB
3	119,576,457	rs568566	AA	AA	AA	AA	AA
3	119,588,320	rs705253	BB	BB	AA	BB	BB
3	119,602,372	rs524438	AA	AA	AA	AA	AA
3	119,603,003	rs518882	BB	BB	AA	AA	AB
3	119,634,579	rs572538	BB	BB	AA	AA	AB
3	119,645,313	rs10511378	AA	AA	AA	AA	AA
3	119,645,381	rs1374767	AA	AA	BB	BB	AB
3	119,645,556	rs571525	AA	AA	AA	AA	AA
3	119,664,546	rs16828526	BB	BB	BB	BB	BB
3	119,665,067	rs1520115	BB	BB	BB	BB	BB
3	119,666,473	rs1949542	AA	AA	BB	BB	AB
3	119,666,634	rs1401953	AA	AA	BB	AA	AA
3	119,666,939	rs7632701	AA	AA	AA	BB	AB
3	122,537,295	rs13067941	AA	AB	AA	AA	AB
3	122,586,192	rs3914164	AA	AA	NoCall	BB	AB
3	122,608,097	rs2331921	AA	AA	BB	BB	AB

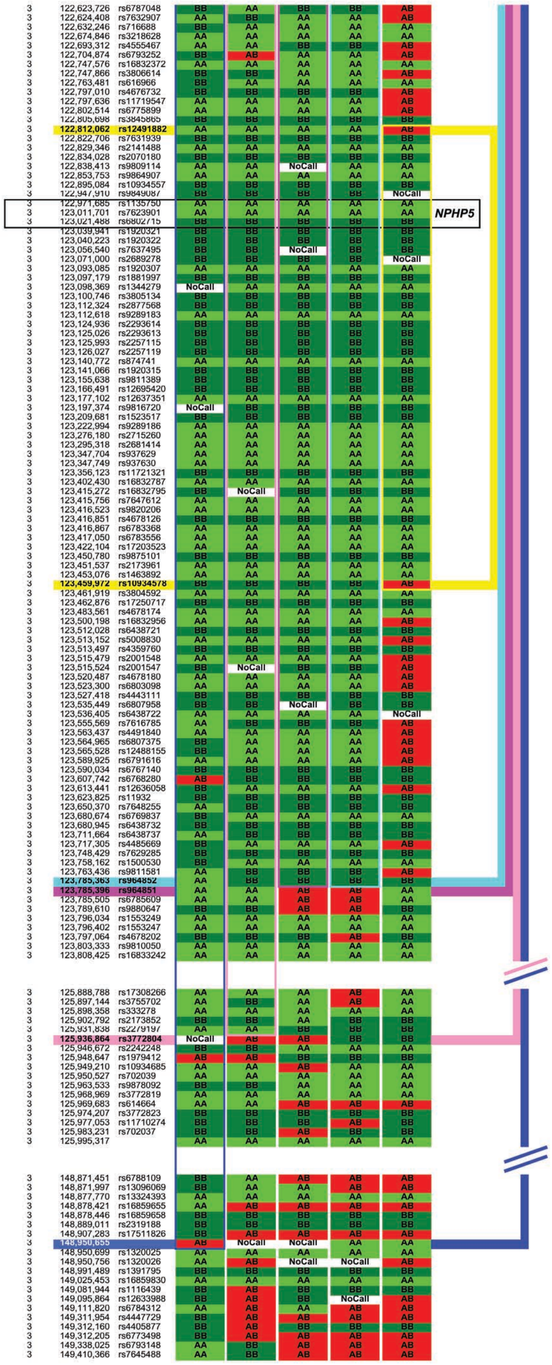

Supplementary Figure 3. The *NPHP5* mutation F142fsX147 occurs on a shared haplotype.
Haplotypes from a 250k SNP array of 5 different families with Senior-Loken syndrome due to the homozygous *NPHP5* mutation F142fsX147 are shown. Homozygous alleles are on green background (light green for "AA", dark green for "BB"). Heterozygous alleles are on red background. "No calls" by the SNP evaluation software BRLMM are on white background. Continous segments of homozygosity (that are partially interrupted by rare false heterozygous allele calls) are encased in colored boxes and delimited by same-color brackets on the right with their heterozygous flanking markers indicated in the same color. The position of the *NPHP5* gene is indicated.
Note that all families within the *NPHP5* region share identical homozygous alleles within haplotypes of varying length. In this way we demonstrate that the *NPHP5* mutation F142fsX147 occurs on a shared haplotype and thereby represents a European "founder" mutation by descent from a common ancestor. The homozygous intervals in the three families from Germany measure 40.6 Mb in F1, 8.2 Mb in F399, 4.5 Mb in A567, and in the two families from Switzerland 5.18 Mb in F408, and 0.65 Mb in F409. The short interval of 0.65 Mb was not detectable as a homozygosity peak (see **Table 1** and **Figure 3**).
