# Supplementary material for: A Systematic Approach to Mapping Recessive Disease Genes in Individuals from Outbred Populations
Source: PLoS Genet. 2009 Jan 23;5(1):e1000353. doi: 10.1371/journal.pgen.1000353 (PMC2621355; doi:10.1371/journal.pgen.1000353)
Supplement: Table S1 — Genetic characteristics of 72 individuals from 54 unrelated families with NPHP or SRNS. (0.23 MB DOC) [file pgen.1000353.s004.doc]

**Supplementary Table S1. Genetic characteristics of 72 individuals from 54 unrelated families with NPHP or SRNS.**

| **Family** | **Individual (individuals calculated together)** | **Disease status** | **Degree of Con-san-guinity** | **Origin** | **SNP density (year run)** | **Gene with homo-zygous mutation** | **Homozygous mutation** | **Number of ZLR peaks/ genome (see Fig. 3)** |
| --- | --- | --- | --- | --- | --- | --- | --- | --- |
| **A1337** | **-1** | **A** | **C** | **Turkey** | **50K** | ***NPHP1*** | **homoz. del** | **60** |
| **F617** | **-1** | **A** | **C** | **Native American** | **250K ('07)** | ***NPHP4*** | **R870fs** | **44** |
| **A1175** | **-1** | **A** | **C** | **Turkey** | **50K** | ***NPHP5*** | **W444X** | **28** |
| **A1125** | **-1** | **A, A** | **C** | **Turkey** | **50K** | ***NPHP3*** | **IVS27+2T>C** | **28** |
| **A1125** | **-2** | **A, A** | **C** | **Turkey** | **50K** | ***NPHP3*** | **IVS27+2T>C** | **17** |
| **A1125** | **(-1, -2)** | **A, A** | **C** | **Turkey** | **50K** | ***NPHP3*** | **IVS27+2T>C** | **5** |
| **A364** | **24** | **A** | **C** | **Pakistan** | **50K** | ***NPHP5*** | **IVS6-1G>A** | **27** |
| **A14** | **-1** | **A** | **C** | **Venezuela** | **250K ('07)** | ***NPHP3*** | **∆G1275** | **10** |
| **A14** | **-2** | **A** | **C** | **Venezuela** | **250K ('07)** | ***NPHP3*** | **∆G1275** | **27** |
| **F3** | **(-1, -3)** | **A, A** | **C** | **Turkey** | **250K ('07)** | ***NPHP4*** | **Q779X** | **7** |
| **F3** | **-1** | **A** | **C** | **Turkey** | **250K ('07)** | ***NPHP4*** | **Q779X** | **20** |
| **F3** | **-3** | **A** | **C** | **Turkey** | **250K ('07)** | ***NPHP4*** | **Q779X** | **25** |
| **F3** | **father** | **U** | **C** | **Turkey** | **250K ('07)** | ***NPHP4*** | **(het. mutation)** | **1** |
| **F3** | **mother** | **U** | **C** | **Turkey** | **250K ('07)** | ***NPHP4*** | **(het. mutation)** | **2** |
| **A1218** | **-1** | **A** | **__** | **USA** | **50K** | ***NPHP1*** | **homoz. del** | **24** |
| **F700** | **(-1, -2)** | **A, A** | **c** | **Turkey** | **10K** | ***NPHP6*** | **G1890X** | **6** |
| **F700** | **-1** | **A** | **c** | **Turkey** | **10K** | ***NPHP6*** | **G1890X** | **20** |
| **F700** | **-2** | **A** | **c** | **Turkey** | **10K** | ***NPHP6*** | **G1890X** | **15** |
| **F1241** | **-1** | **A** | **C** | **Kuwait** | **250K ('07)** | ***NPHP4*** | **R59X** | **16** |
| **F799** | **24** | **A** | **C** | **Arab** | **10K** | ***AHI1*** | **N791X** | **15** |
| **F543** | **(-1, -2)** | **A, A** | **C** | **Turkey** | **250K ('06)** | ***NPHP10*** | **N311fsX315** | **4** |
| **F543** | **-1** | **A** | **C** | **Turkey** | **250K ('06)** | ***NPHP10*** | **N311fsX315** | **15** |
| **F543** | **-2** | **A** | **C** | **Turkey** | **250K ('06)** | ***NPHP10*** | **N311fsX315** | **13** |
| **A8** | **-1** | **A** | **c** | **Turkey** | **250K ('07)** | ***NPHP2*** | **R603X** | **14** |
| **A1371** | **-1** | **A** | **c** | **Arab** | **250K ('06)** | ***MKS3*** | **S630P** | **12** |
| **A1538** | **-1** | **A** | **c** | **Arab** | **250K ('06)** | ***NPHP5*** | **IVS11-1G>C** | **9** |
| **A1538** | **-2** | **A** | **c** | **Arab** | **250K ('07)** | ***NPHP5*** | **IVS11-1G>C** | **12** |
| **A1538** | **-3** | **A** | **c** | **Arab** | **250K ('07)** | ***NPHP5*** | **IVS11-1G>C** | **10** |
| **A1538** | **(-1, -2)** | **A, A** | **c** | **Arab** | **250K ('07)** | ***NPHP5*** | **IVS11-1G>C** | **4** |
| **A1538** | **(-1, -3)** | **A, A** | **c** | **Arab** | **250K ('07)** | ***NPHP5*** | **IVS11-1G>C** | **5** |
| **A1538** | **(-2, -3)** | **A, A** | **c** | **Arab** | **250K ('07)** | ***NPHP5*** | **IVS11-1G>C** | **3** |
| **A1538** | **(-1, -2, -3)** | **A, A, A** | **c** | **Arab** | **250K ('07)** | ***NPHP5*** | **IVS11-1G>C** | **2** |
| **A646** | **-1** | **A** | **__** | **Germany** | **250K ('06)** | ***NPHS2*** | **R138Q** | **11** |
| **F944** | **(-1, -2)** | **A, A** | **C** | **Turkey** | **10K** | ***NPHP6*** | **G1890X** | **2** |
| **F944** | **-1** | **A** | **C** | **Turkey** | **10K** | ***NPHP6*** | **G1890X** | **8** |
| **F944** | **-2** | **A** | **C** | **Turkey** | **10K** | ***NPHP6*** | **G1890X** | **11** |
| **F585** | **-1** | **A** | **C** | **Turkey** | **250K ('06)** | ***TMEM67*** | **C615R** | **10** |
| **F1158** | **-1** | **A** | **c** | **German** | **250K ('07)** | ***NPHP1*** | **G343R** | **9** |
| **F4** | **(-1, -2)** | **A, A** | **C** | **Turkey** | **10K** | ***NPHP6*** | **2218-2222_del_ccagATAGA** | **4** |
| **F4** | **-1** | **A** | **C** | **Turkey** | **10K** | ***NPHP6*** | **2218-2222_del_ccagATAGA** | **9** |
| **F4** | **-2** | **A** | **C** | **Turkey** | **10K** | ***NPHP6*** | **2218-2222_del_ccagATAGA** | **5** |
| **A166** | **-1** | **A** | **C  1st cousin** | **USA** | **250K ('06)** | ***RPGRIP1L*** | **T615P** | **8** |
| **F601** | **-1** | **A** | **c** | **Germany** | **250K ('06)** | ***NPHP9*** | **H425Y** | **8** |
| **A1934** | **-1** | **A** | **c** | **Hispanic** | **250K ('07)** | ***NPHS2*** | **Y162X** | **8** |
| **A1357** | **(-1, -2)** | **A, A** | **c** | **Turkey** | **250K ('06)** | ***NPHS1*** | **Ex24_3243_3250ins** | **2** |
| **A1357** | **-1** | **A** | **c** | **Turkey** | **250K ('06)** | ***NPHS1*** | **Ex24_3243_3250ins** | **8** |
| **A1357** | **-2** | **A** | **c** | **Turkey** | **250K ('06)** | ***NPHS1*** | **Ex24_3243_3250ins** | **7** |
| **A825** | **-1** | **A** | **__** | **Slavic** | **250K ('06)** | ***NPHS2*** | **R138Q** | **7** |
| **A1680** | **-1** | **A** | **c** | **Turkey** | **250K ('07)** | ***NPHS1*** | **A850fsX873** | **7** |
| **A1367** | **(-1, -2)** | **A, A** | **c** | **Arab** | **250K ('07)** | ***NPHP8*** | **A695P** | **2** |
| **A1367** | **-1** | **A** | **c** | **Arab** | **250K ('07)** | ***NPHP8*** | **A695P** | **6** |
| **A1367** | **-2** | **A** | **c** | **Arab** | **250K ('07)** | ***NPHP8*** | **A695P** | **4** |
| **A131** | **(-1, -2, -3)** | **A, A, A** | **c** | **Lapland** | **50K** | ***NPHP10*** | **G453C** | **1** |
| **A131** | **(-1, -2)** | **A, A** | **c** | **Lapland** | **50K** | ***NPHP10*** | **G453C** | **4** |
| **A131** | **(-2, -3)** | **A, A** | **c** | **Lapland** | **50K** | ***NPHP10*** | **G453C** | **3** |
| **A131** | **(-1, -3)** | **A, A** | **c** | **Lapland** | **50K** | ***NPHP10*** | **G453C** | **2** |
| **A131** | **-1** | **A** | **c** | **Lapland** | **50K** | ***NPHP10*** | **G453C** | **4** |
| **A131** | **-2** | **A** | **c** | **Lapland** | **50K** | ***NPHP10*** | **G453C** | **3** |
| **A131** | **-3** | **A** | **c** | **Lapland** | **50K** | ***NPHP10*** | **G453C** | **5** |
| **F624** | **(-1, -2)** | **A, A** | **c** | **Arab** | **250K ('07)** | ***NPHP3*** | **IVS26+1G>A** | **2** |
| **F624** | **-1** | **A** | **c** | **Arab** | **250K ('07)** | ***NPHP3*** | **IVS26+1G>A** | **5** |
| **F624** | **-2** | **A** | **c** | **Arab** | **250K ('07)** | ***NPHP3*** | **IVS26+1G>A** | **4** |
| **F624** | **father** | **U** | **c** | **Arab** | **250K ('07)** | ***NPHP3*** | **(het. mutation)** | **0** |
| **F624** | **mother** | **U** | **c** | **Arab** | **250K ('07)** | ***NPHP3*** | **(het. mutation)** | **0** |
| **F1405** | **-1** | **A** | **C** | **Turkey** | **50K** | ***NPHP1*** | **IVS18+1G>A** | **4** |
| **F704** | **-2** | **A** | **c** | **Turkey** | **250K ('07)** | ***NPHP4*** | **Q1050fs** | **4** |
| **F88** | **-3** | **A** | **__** | **Italy** | **10K** | ***NPHP4*** | **R488X** | **4** |
| **A237** | **(-1, -2)** | **A, A** | **__** | **Slavic** | **250K ('06)** | ***NPHS2*** | **R138Q** | **2** |
| **A237** | **-1** | **A** | **__** | **Slavic** | **250K ('06)** | ***NPHS2*** | **R138Q** | **3** |
| **A237** | **-2** | **A** | **__** | **Slavic** | **250K ('06)** | ***NPHS2*** | **R138Q** | **4** |
| **F60-61** | **-1** | **A** | **__** | **France** | **250K ('07)** | ***NPHP4*** | **R658X** | **2** |
| **F61-60** | **-2** | **A** | **__** | **France** | **250K ('07)** | ***NPHP4*** | **R658X** | **4** |
| **F61 + F60** | **(-1,-2)** | **A, A** | **__** | **France** | **250K ('07)** | ***NPHP4*** | **R658X** | **1** |
| **F138** | **-1** | **A** | **__** | **Germany** | **250K ('07)** | ***NPHP8*** | **T615P** | **3** |
| **F456** | **-1** | **A** | **__** | **Italy** | **250K ('07)** | ***NPHP4*** | **T946A** | **3** |
| **F50** | **(-1, -3)** | **A, A** | **__** | **Germany** | **250K ('07)** | ***NPHP5*** | **H506fs** | **1** |
| **F50** | **-1** | **A** | **__** | **Germany** | **250K ('07)** | ***NPHP5*** | **H506fs** | **3** |
| **F50** | **-3** | **A** | **__** | **Germany** | **250K ('07)** | ***NPHP5*** | **H506fs** | **2** |
| **F50** | **father** | **U** | **__** | **Germany** | **250K ('07)** | ***NPHP5*** | **-** | **0** |
| **F50** | **mother** | **U** | **__** | **Germany** | **250K ('07)** | ***NPHP5*** | **-** | **0** |
| **F1183** | **21** | **A** | **__** | **Sweden** | **250K ('07)** | ***NPHP4*** | **T1004fs** | **3** |
| **A762** | **-1** | **A** | **__** | **Germany** | **250K ('06)** | ***NPHP8*** | **C633R** | **2** |
| **F53** | **-1** | **A** | **__** | **Germany** | **250K ('07)** | ***NPHP5*** | **L149fs** | **2** |
| **F53** | **father** | **U** | **__** | **Germany** | **250K ('07)** | ***NPHP5*** | **-** | **7** |
| **F53** | **mother** | **U** | **__** | **Germany** | **250K ('07)** | ***NPHP5*** | **-** | **1** |
| **F1298** | **-2** | **A** | **__** | **Germany** | **250K ('07)** | ***NPHP5*** | **H506fs** | **2** |
| **F1298** | **father** | **U** | **__** | **Germany** | **250K ('07)** | ***NPHP5*** | **-** | **0** |
| **F1298** | **mother** | **U** | **__** | **Germany** | **250K ('07)** | ***NPHP5*** | **-** | **1** |
| **A567** | **-1** | **A** | **__** | **Germany** | **250K ('07)** | ***NPHP5*** | **F142fs** | **2** |
| **A7** | **-1** | **A** | **__** | **Portugal** | **250K ('07)** | ***NPHP2*** | **L493S** | **2** |
| **F54** | **-1** | **A** | **__** | **Germany** | **250K ('07)** | ***NPHP5*** | **H506fs** | **2** |
| **A1338** | **-1** | **A** | **__** | **Israel/Slavic** | **50K** | ***NPHP1*** | **homoz. del** | **2** |
| **F399** | **(-1, -2)** | **A, A** | **__** | **Germany** | **250K ('07)** | ***NPHP5*** | **F142fs** | **1** |
| **A1730** | **-1** | **A** | **__** | **Germany** | **250K ('07)** | ***NPHS2*** | **R138Q** | **1** |
| **A1730** | **-2** | **A** | **__** | **Germany** | **250K ('06)** | ***NPHS2*** | **R138Q** | **1** |
| **A1730** | **-2** | **A** | **__** | **Germany** | **250K ('06)** | ***NPHS2*** | **R138Q** | **1** |
| **F30** | **-1** | **A** | **10 gene-rations** | **Germany** | **250K ('07)** | ***NPHP4*** | **c.3272delT; p.fsX1121** | **1** |
| **F30** | **-3** | **A** | **10 gene-rations** | **Germany** | **250K ('07)** | ***NPHP4*** | **c.3272delT; p.fsX1121** | **0** |
| **F408** | **-1** | **A** | **__** | **Switzer-land** | **250K ('07)** | ***NPHP5*** | **F142fs** | **1** |
| **F409** | **-1** | **A** | **__** | **Switzer-land** | **250K ('07)** | ***NPHP5*** | **F142fs** | **0** |
| **A159** | **-1** | **A** | **__** | **Czech** | **250K ('07)** | ***NPHS2*** | **R138Q** | **0** |
| **A1686** | **-1** | **A** | **__** | **Switzer-land** | **250K ('06)** | ***NPHS2*** | **R138Q** | **0** |
| **A1685** | **-1** | **A** | **__** | **Germany** | **250K ('07)** | ***NPHP8*** | **Y817C** | **0** |
| **A887** | **-1** | **A** | **__** | **Germany** | **250K ('06)** | ***NPHS2*** | **R138Q** | **0** |
| **F190** | **-1** | **A** | **__** | **Germany** | **250K (’07)** | ***MKS3*** | **Compound heterozygous M232T; C615R** | **0** |

A, affected by disease; U, unaffected by disease; C, consanguinity known to be present; c, consanguinity not known but originating from population with frequent consanguinity;-- , originating from outbred population.
